# Zoonotic Diseases and Phytochemical Medicines for Microbial Infections in Veterinary Science: Current State and Future Perspective

**DOI:** 10.3389/fvets.2018.00166

**Published:** 2018-07-24

**Authors:** Bora Shin, Woojun Park

**Affiliations:** Laboratory of Molecular Environmental Microbiology, Department of Environmental Sciences and Ecological Engineering, Korea University, Seoul, South Korea

**Keywords:** adjuvant, alternative medicines, veterinary, zoonotic infection, plant extract, phytotherapy

## Abstract

Diseases caused by bacterial infections in small-scale and industrial livestock are becoming serious global health concern in veterinary science. Zoonotic bacteria, including *Staphylococcus, Campylobacter*, and *Bartonella* species, that infect animals and humans cause various illnesses, such as fever, diarrhea, and related complications. Bacterial diseases in animals can be treated with various classes of antibiotics, including fluoroquinolones, beta-lactams, aminoglycosides, and macrolides. However, the overuse and misuse of antibiotics have led to drug resistance in infectious agents, e.g., methicillin-resistant *Staphylococcus*; this hampers the treatment of infections in livestock, and such problems are increasing worldwide. Dietary phytochemicals and herbal medicines are useful and viable alternatives to pharmaceuticals because they are economical, effective, non-resistance-forming, renewable, and environmentally friendly. They are small molecules with high structural diversity that cause selective stress to or stimulation of resident microbiota, consequently causing an abundance of such microorganisms; thus, they can be used in preventing various diseases, ranging from metabolic and inflammatory diseases to cancer. In addition, the antioxidant effects of phytochemicals prevent substantial losses in the livestock industry by increasing animal fertility and preventing diseases. Potentially effective plant extracts could be used in combination with antibiotics to decrease the required dose of antibiotics and increase their effectiveness. This strategy can help avoid the side effects of chemical antimicrobials and allow the effective use of phytochemicals for treating diseases. Furthermore, phytochemicals are considered as potential alternatives to antibiotics because of their economical, non-resistance-forming and environmentally friendly properties. Flavonoids such as resveratrol, epigallocatechin gallate, and phenols such as galangin, puerarin, and ursolic acid are proven to be effective as antimicrobial agents. This review provides invaluable information about the types of microbial infections in animals and the current knowledge on phytotherapeutic agents classified by their mode of actions. It also provides insights into potential strategies for effectively treating animal infections using phytochemicals.

## Introduction

The health of humans and animals has been threatened by increasing resistance to antibiotics, environmental pollution, and the development of chronic diseases (1). It is necessary to understand and use the concept of One Health to effectively control and prevent diseases in the human–animal interface. The concept of One Health is currently advancing with the emergence and spread of epizootics, zoonoses, and epidemics, whereas the risks of pandemics have become an increasing critical challenge (2). Antimicrobial agents have seen general use in human and veterinary medicine for >50 years and have shown tremendous health benefits (3). However, the misuse and overuse of antibiotics generate selective evolutionary pressures that increase the chance of survival of antibiotic-resistant bacteria, which puts individuals at risk of becoming infected by drug-resistant bacteria (4). This development of antibiotic resistance renders the antimicrobial therapies ineffective, thus posing a serious public health threat.

In the last decade, the interest in phytotherapy has increased because of decline in the field of antibiotic research and increased concerns about the spread of antibiotic resistance (5). The use of plants as a remedy in traditional medicine is as old as humankind. Understanding the ingredients of plant defense systems is essential to gain benefit from their abundant phytochemical compounds, which can be used as medications for clinical application (6). Plants are a rich source of bioactive substances that have a protective effect against harmful microorganisms; thus, they are being extensively researched as promising materials that can be used in the development of antibiotics and alternative medicines (6).

The World Health Organization (WHO) Traditional Medicine Strategy 2014–2023 was established to assess traditional and complementary medicine, including herbs and other plant materials (7). Nonetheless, the mechanisms of action of most phytotherapeutic chemicals are not fully understood. However, their synergistic antimicrobial activity is generally assumed to damage the bacterial membrane by lipophilic compounds or reduce cell division by DNA synthesis inhibition (8).

Phytotherapy has become an important new concept in healthcare research that was prompted by the need for alternatives to ineffective conventional antibiotics. The development of novel and efficient extraction techniques has led to renewed and increasing interest in plant-derived bioactive compounds (8). Examples of phytotherapeutic drugs include oleanolic acid, which is used as a natural adjuvant for aminoglycosides as it increases their membrane permeability (9). Most plant-derived compounds have weaker antibiotic activity than the common chemicals produced by bacteria and fungi. This review attempts to provide knowledge about zoonotic diseases and insight into the rich variety of antimicrobial secondary metabolites (i.e., phytochemicals) from plants that can be applied in the treatment of zoonoses.

### Risk of zoonotic bacterial infection and veterinary health impact

**Figure 1:**
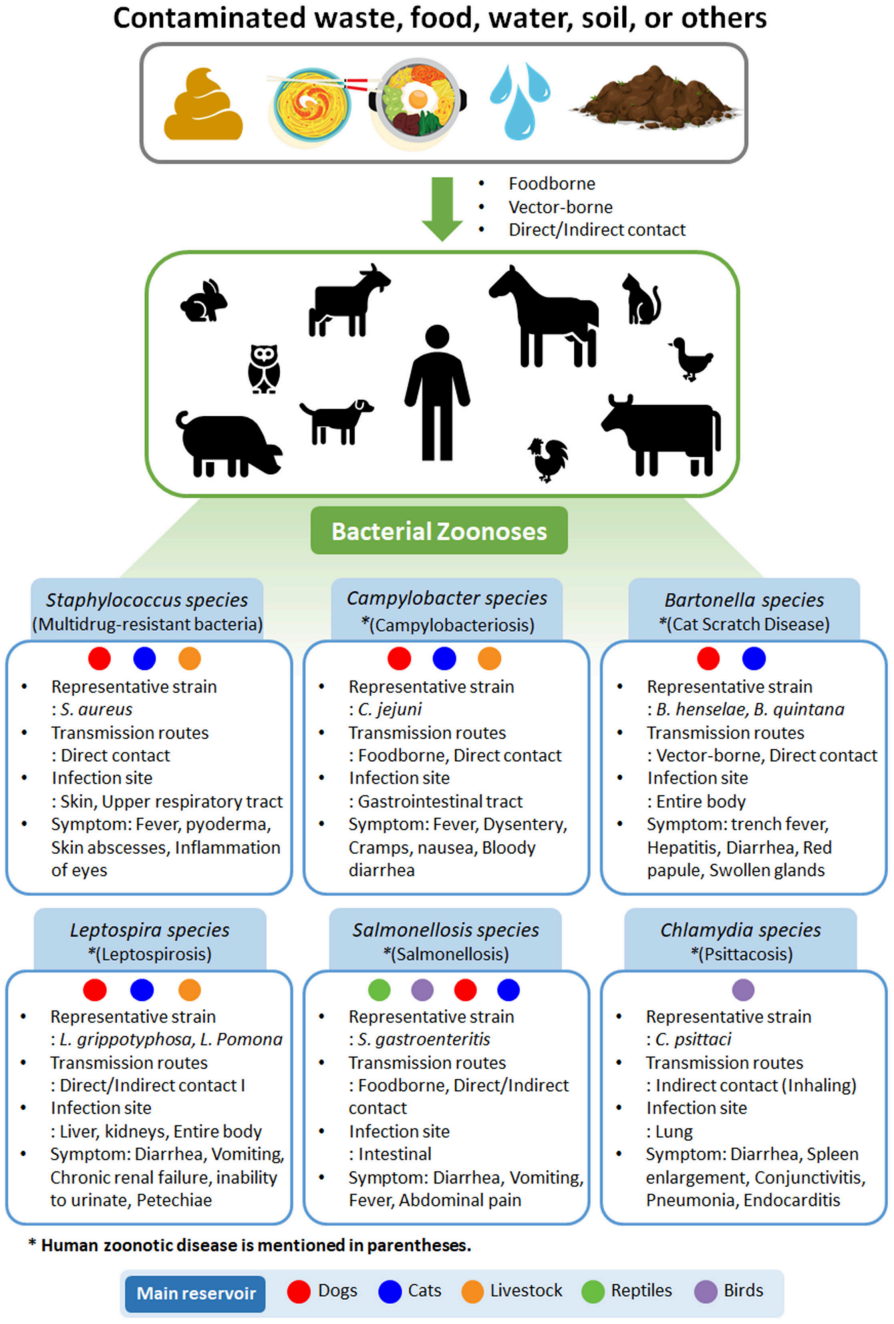
Types of bacterial zoonoses in humans and animals. Representative strain, transmission routes, infection site, and symptoms are summarized. Main reservoirs are classified as dogs, cats, livestock, reptiles, and birds. Disease names are given in parentheses.

A zoonosis is an infectious disease that can be transmitted from animals to humans. Currently 61% of pathogens known to affect humans are zoonotic (10). Most emerging infectious diseases considered to be serious public health problems have zoonotic origins (11), and approximately three-quarters have originated from wild animals (12). Zoonotic pathogens can be transmitted by close contact with an animal, generally through inhalation, ingestion, or other routes that contaminate mucous membranes and damaged or, in some cases, intact skin (12, 13). Aerosol-mediated transmission is occasional, particularly in confined spaces. Fomites can transmit some agents, and the likelihood of this route correlates with the persistence of the organism in the environment. Transmission of some organisms occurs via ingestion of contaminated food or water, and such organisms may infect large number of people. Sources of zoonotic pathogens in foodborne diseases include undercooked meat or other animal tissues, seafood, and invertebrates, as well as unpasteurized milk and dairy products and contaminated vegetables (14). Insects serve as important biological or mechanical vectors in transmitting some organisms (15). In this review, we have summarized representative bacterial zoonotic infections (Figure 1).

In veterinary medicine, *S. aureus* is a gram-positive bacterium that commonly exists on the skin and mucous membranes of healthy humans and animals. However, it has also been recognized as a significant opportunistic pathogen in chickens and farmed rabbits and has been reported to cause mastitis in dairy-producing animals (16). It is most commonly isolated from staphylococcosis cases, but species such as *S. hyicus* have been reported as causative agents of osteomyelitis in turkeys and of exudative epidermitis in pigs (13, 15, 16). The symptoms of staphylococcosis vary depending on the site and route of inoculation and can involve the bones, joints, tendon sheaths, skin, sternal bursa, navel, and yolk sac. Immunocompromised birds are also more prone to staphylococcal infections (16). Staphylococcosis can be treated with antibiotics; however, methicillin-resistant *S. aureus* has recently emerged (17). In staphylococci, methicillin resistance is mediated by penicillin-binding proteins (i.e., *mec*A and *mec*C), which are suggested to be of animal origin and have demonstrated low affinity for beta-lactams (18–20). Common antibiotics used to treat *Staphylococcus* infections are penicillin, erythromycin, lincomycin, and spectinomycin (19). Proper management to prevent injury and immunocompromised conditions in poultry facilitate the prevention of staphylococcosis (21).

In the poultry industry, campylobacteriosis is also a common disease transmitted from other livestock, e.g., farmed cattle, swine, or poultry, with staphylococcosis (22). *Campylobacter jejuni* and *C. coli* are representative pathogens that are a major cause of human gastroenteritis (22). Their predominant ecological niche is the gastrointestinal tract of a wide range of domesticated and wild vertebrates, and zoonotic transmission from animals to people via meat, especially chicken, is a food safety issue (23). *Campylobacter* spp. are also commonly isolated from a wide variety of birds, including waterfowl, raptors, crows, and pigeons, that pollute the habitats of grazing animals. In addition, insects and rodents, such as flies, rats, and mice, have been reported to transmit *C. jejuni* (22). Other species, such as *C. lari, C. helveticus*, and *C. upsaliensis*, have also been isolated from patients with diarrheal disease but are reported less frequently. Campylobacteriosis is most commonly treated with azithromycin, levofloxacin, clindamycin, aminoglycodies, fluoroquinolones (e.g., nalidixic acid), and cephalosporins (e.g., cephalothin). However, the emergence of resistance to cephalosporins has rapidly increased over the last 10 years (23). Thus, it is necessary to find new drugs or supplements to reduce resistance to antibiotics.

According to the American Pet Products Association, the pet industry has expanded steadily at an average of 4% per year over the last two decades (24). As a result, interest and research into pet-related zoonotic diseases is increasing. Common cat scratch disease is primarily caused by *Bartonella henselae* (25). Although this *Bartonella* sp. can cause many types of animal infections, pets have been identified as notable reservoirs for human infection, implying a potentially high risk of humans (25). Generally, *B. bacilliformis* and *B. quintana* are considered human-specific pathogens, but several zoonotic *Bartonellae* spp. specific to diverse animal hosts can also infect humans as incidental hosts (25, 26). Pathogenic *Bartonella* spp. are endotheliotropic bacteria with a distinctive mechanism for invasion into host cells involving the injection of peptides and transport of bacterial DNA into the cells; these species can move by infecting macrophages (26). Recently, significant associations have been found between amino acid alleles of Toll-like receptors and susceptibility to infection with the blood pathogenic and clinically isolated *Bartonella* sp. Cat fleas, sand flies, human body lice, and many other flea species can transmit certain *Bartonella* spp. (14, 26). It has been reported that in addition to pets, other animals, including rodents, cattle, deer, and sheep, can spread *Bartonella* infection through flies or deer keds (*Lipoptena cervi*) (27). Most cases of cat scratch disease get cured without treatment; however, some immunocompromised patients can present complications from disseminated diseases (25). *Bartonella* spp. can cause acute or chronic infection with vascular proliferative or suppurative manifestations. Blood culture-negative endocarditis and bacteremia can also be induced by a spectrum of *Bartonella* spp. in canine and human patients (15). Numerous antibiotics, including azithromycin, penicillin, tetracyclines, cephalosporins, and aminoglycosides, are effective against *Bartonella* infection (25, 26). Doxycycline, amoxicillin, enrofloxacin, and rifampin given for a long duration (more than 4 weeks) may effectively reduce the level of bacteremia in an infected cat or dog, although there is a risk of side effects (25).

*Leptospira* is an endemic bacterium in many domestic and wild animals that spreads through urine (28) and causes leptospirosis in humans and animals through contact with urine-contaminated water or soil. Thus, leptospirosis occurs in both humans and animals, including livestock and marine mammals (28). It is one of the most common and severe human infections worldwide. In addition, it can cause a variety of symptoms in animals and humans, some of which can be mistaken for other diseases. Further, some infected animals and humans may act as a source of infection without symptoms (29). Leptospirosis is generally limited to developing countries and has been reported as an imported disease in industrialized countries (28). Leptospirosis can cause fever, meningitis, kidney damage, respiratory distress, liver failure, and even death if it causes severe complications (30). Coinfections with common endemic bacteria that cause acute febrile illnesses (e.g., salmonellosis), can be a diagnostic dilemma if symptoms overlap (31). For example, *Salmonella*^1^ can enter the bloodstream and infect the spine through gastric mucosal vasculitis, which may be caused by *Leptospira* (30).

Human salmonella infections are typically caused by direct or indirect exposure to contaminated food or various host species, including dogs, cats, livestock, domestic poultry, and rodents (23). There are over 2,300 subtypes of the *Salmonella enterica*, including serovars *Enteritidis, Agbeni*, and *Typhimurium*, that can cause asymptomatic, mild clinical, or fulminant bacteremia/septicemia, and endotoxemic infections (23). According to a report by the Centers for Disease Control and Prevention, *Salmonella* infection in humans results in a diarrheal illness that is responsible for 450 deaths in the United States annually (32). *Salmonella* is an opportunistic pathogen that causes a wide range of infectious symptoms, including food poisoning, typhoid fever, enteric fever, and gastroenteritis, depending on the immunity of the host, the infection dose, and the virulence of the strain (30, 31).

Although considerable research is being conducted on zoonotic bacteria, there is still a lack of scientific knowledge about the distribution and infection cycles of *Chlamydia* spp. compared with other zoonotic bacteria. The respiratory illness psittacosis caused by *Chlamydia* sp. in humans and animals was first reported approximately 100 years ago (33); however, only 218 reports on illness were found when searched using “Chlamydial, Zoonoses” in the NCBI database. With the recent occurrences of severe acute respiratory syndrome and avian influenza, studies on *Chlamydia* infections and zoonoses have increased rapidly, and 53 related papers have been published since 2015. Transmission of *C. psittaci* infection primarily occurs through inhalation of contaminated aerosols from infected birds (34). C. trachomatis infection causes reproductive complications, and *C. pneumoniae* causes respiratory infections and atypical pneumonia in both humans and animals. *C. suis* infection is associated with diarrhea and failure to gain weight in domestic swine (33, 34). In general, most people infected with *Chlamydia* do not show any symptoms (34). However, in female animals with weak immune systems, pathogens can be transferred to the womb, causing pelvic inflammatory disease, a major cause of ectopic pregnancy and female infertility (33, 34). *Chlamydia* infections is most commonly treated by antibiotics, such as azithromycin and doxycycline (33). However, with antibiotic resistance of pathogenic bacteria becoming a major problem worldwide, there is a need to develop safe drugs with few side effects to treat infection in both humans and animals. In addition, the overuse and misuse of antibiotics are associated with both human and animal immune systems. The collapse of gut microbial ecosystems due to antibiotics could cause undesired negative effects, such as altered physiology of the body or susceptibility to infectious diseases (35, 36).

### Antimicrobial phytochemicals in veterinary diseases

Bacteria get resistant to antibiotics rapidly; thus, there is an urgent need to develop effective antimicrobial compounds or to find adjuvants that promote antibiotic function. Plant therapy is one of the oldest medical fields traditionally based on empiricism and is considered the largest source of new antibiotics to address antibiotic resistance (5). Commonly, synthetic medicines are expensive and damage the intestinal microbial balance due to their toxicity; by contrast, herbal antibacterial compounds are relatively non-toxic, inexpensive, and environmentally friendly (37). Microbiota is involved in the control of metabolism in humans and animals via protection against pathogens, enhancement of immune systems, and production of vitamins and short-chain fatty acids (35). It is known to have an important influence on human and animal health through interaction with the immune system. In the past, medical herbs were used as medicines to prevent and/or treat infection. For example, in ancient China and Egypt, records of medicinal plant use for health maintenance and disease prevention since 3,000 BC have been found (38). In general, the ratio of *Firmicutes* to *Bacteroidetes* in gut microbiome is highly relevant to the immune system and can vary dramatically depending on the health (35) and age (middle or old age) of the individual (36). Herbal medicine interacts with the gut microbiome and alters metabolites, including short-chain fatty acids, bile acids, and lipopolysaccharides (35).

Herbal remedies are generally characterized by wide therapeutic indexes (37), and they consist of multicomponent mixtures acting as multi-target drugs with pleiotropic effects. In animals, self-medication remains a controversial subject because the evidence is mostly anecdotal (39). Unlike the popular belief that most drugs are synthetic in origin, many important medicines, such as cocaine and atropine, are natural products of plant origin (40). Plants are used as natural resources in the development of new drugs, over the counter drugs, and nutraceuticals. In recent years, the consumption of botanical supplements has increased globally due to their relatively low cost and the need to reduce antibiotic overuse (38). For example, terpenoids and phenolic compounds extracted from plants have been extensively studied for their immunity-enhancing effects and antimicrobial activities against a broad range of infectious microorganisms (Table 1). As shown in Table 1, typical phytochemicals with antioxidant activity include monoterpenes (aromatic plants), diterpenes (berries and essential oils), triterpenes (olive oil and leaves), tetraterpenes (colored fruits and vegetables), sesquiterpenes (essential oils), and polyphenols (from tea and grapes).

**Table 1:** Mode of actions and antibacterial target strains of functional phytochemicals.

**Class**		**Source(s)**	**Compounds**	**Mode of action**	**Reported target bacteria**	**References**
Terpenoids	Monoterpenes	Essential oils of several aromatic plants	Palmarosa oil (*Cymbopogon martinii*), cinnamon oil, geraniol, carvacrol, thymol, eugenol	Antimicrobial, pest control, antioxidant, anti-inflammatory, anti-fungal	*Escherichia coli, Salmonella typhimurium, Streptococcus pneumoniae, S. pyogenes, Staphylococcus aureus*	(41–43)
	Diterpenes	Essential oil from berries, traditional Chinese medicine (*Euphorbiaceae, Lamiaceae*, and *Taxaceae* family)	Andrographolide (*Andrographis paniculata*), oridonin	Antimicrobial, anti-inflammatory, anticancer, antioxidant	*Mycobacterium tuberculosis*	(41, 44)
	Triterpenes	Olives, olive tree leaves, and virgin olive oil	Oleanolic acid, ursolic acid, ginsenoside, gypenosides, betulinic acid, rotundic acid, amyrin, saponins, tirucallane-type of *Eurycoma longifolia*	Antioxidant, antimicrobial, antimalaria, anticancer, treatment of chronic diarrhea	*Acinetobacter baumannii, St. aureus, Bacillus cereus, Pseudomonas aeruginosa*	(9, 41, 45–47)
	Tetraterpenes	Colored fruits, green leafy vegetables, microalgae	Astaxanthin, β-carotene, fucoxanthin	Antioxidant, anti-inflammatory, treatment of cardiovascular disease, anticancer		(41, 48, 49)
	Sesquiterpenes	Odor of essential oils	α-Bisabolol, dehydrocostuslactone	Antioxidant		(41, 50)
Phenolic compounds	Polyphenols	Berries, needles of *Cedrus deodara*, mushroom	Anthocyanins, cyanidins, quercetin, myricetin, rutin, tannins, CHQA	Enhancing the health of the gut (impact on gut microbiome), antioxidant, anti-adhesion	*E. coli, St. aureus, Neisseria gonorrhoeae, Pasteurella multocida*	(18, 51, 52)
		Bran or hull	Benzoic acid, cinnamic acid, benzaldehydes	Antifungal, antimicrobial	*A. bauamnnii, Candida albicans, Campylobacter jejuni, E. coli, Listeria monocytogenes, Sa. enterica*,	(5, 23, 53)
		Chili peppers, oats	Capsaicinoids, avenanthramides	Pest control, antioxidant, anti-inflammatory, inhibition of LDL oxidation, thermal hyperalgesia, beneficial in obesity	*Helicobacter pylori, S. pyogenes, St. aureus, St. typhimurium, P. aeruginosa*	(53–56)
		Grapes, red wine, sesame, green tea, cocoa (flovanols)	Resveratrol, (–)-epigallocatechin gallate, ellagic acid, lignans, curcumin, caffeic acid, gallic acid	Anti-inflammatory, anti-colone cancer, antioxidant, Enhancing the health of the gut, antimicrobial	*Sa. Typhimurium, St. aureus, H. pylori, Clostridium* spp., *Bacteroides* spp.	(51–53, 57)

Phytochemicals, which are bioactive plant components with an amphipathic or hydrophobic structure, are known to exhibit pharmacological effects on interaction with microbial membranes, such as changes in membrane permeability and microviscosity (37). Several phytochemicals have been reported to have antitumor, anti-inflammatory, and antioxidant effects as well as antimicrobial (microbial growth-inhibiting, antibacterial, and antifungal) activities (45, 48, 54). The molecular mechanism of action of plant compounds has been conventionally interpreted or theorized based on their effects on receptors, enzymes, ion channels, transporters, and biological pathways (44, 58, 59). However, plant compounds are responsible for a wide spectrum of pharmacological activities, thereby making it difficult to explain a single action on a particular target (37, 51). Typically, terpenoids and phenolic compounds are the most commonly known antimicrobial plant components (51).

Reportedly, terpenoids have exhibited cancer preventive effects and cytotoxic effects on tumor cells in preclinical animal models (41). Monoterpenes, such as palmarosa oil, cinnamon oil, geraniol, carvacrol, eugenol, and thymol, are present in aromatic plants, fruits, vegetables, and herbs (60). Essential oils, which are terpenoids, are powerful antioxidants effective at scavenging free radicals. Palmarosa oil from *Cymbopogon martinii* is primarily used in skin care and treatment of throat infection and has also demonstrated the highest antimicrobial activity against *S. aureus* and *Escherichia coli* (42). In one study, a checkerboard assay revealed synergistic effect of cinnamon oil with other antimicrobial agents, such as chlorhexidine, triclosan, and gentamicin (61). Cinnamon oil exhibits antimicrobial activity on both planktonic and attached cells of clinically isolated *S. epidermidis* and *S. aureus* strains (62). Interestingly, cinnamon oil has been shown to increase the effectiveness of gentamicin and chlorhexidine as well as the ability to detach and kill *S. epidermidis* biofilms (61, 62). Geraniol from *Pelargonium graveolens* is used as a flavoring agent and stimulant (63) and exhibits significant growth-inhibitory effects against *S. aureus, Bacillus subtilis, Klebsiella pneumoniae*, and *E. coli* (43). The phenolic monoterpenes carvacrol and thymol are major components of the essential oils of *Origanum* and *Thymus* species (64). Carvacrol and thymol have been extensively tested as antimicrobial compounds against pathogens, including *B. cereus, E. coli* O157:H7, *Enterococcus faecalis, Listeria monocytogenes, Sa. typhimurium, S. aureus, Pseudomonas fluorescens*, and *Vibrio cholerae* (43, 64). In one study, the live/dead assay of *Streptococcus pyogenes* demonstrated dead cells 1 h after the incubation of live cells with carvacrol (43). Eugenol and isoeugenol are components of essential oils and are widely studied. They showed synergistic effect with most antibiotics against *E. coli, E. aerogenes, P. vulgaris, P. aeruginosa* and *S. typhimurium*, among which the most obvious are ampicillin, polymyxin B, norfloxacin, tetracycline, rifampicin, and vancomycin (64, 65).

Antimycobacterial activity of andrographolide, a natural diterpenoid, has been demonstrated using luciferase reporter phage assay (66), confirming that andrographolide exhibits antimicrobial activity by targeting the aminoglycoside 2′-N-acetyltransferase in *Mycobacterium tuberculosis* (66). It is widely used in traditional Chinese medicine and shows a wide range of biological effects, including anti-cancer and anti-inflammatory effects (44). In addition, it exhibits weak antimicrobial activity against *S. aureus* and *B. subtilis*. The specific antimicrobial mechanisms of most phytochemicals are not known; however, triterpenes, including oleanolic and ursolic acids, have been shown to exhibit antibacterial activity as adjuvants of aminoglycoside by changing membrane permeability and proton-motive force against *Acinetobacter baumannii* (9). Other triterpenoids, such as betulinic acid, rotundic acid, and amyrin, have shown anti-staphylococcal activities. Although antibacterial effects of phytochemicals are generally weaker than those of antibiotics, phytochemicals can be synergistically used with antibiotics to develop different antibacterial mechanisms or pathways to produce antimicrobial effects against *S. aureus, B. cereus*, and *P. aeruginosa* (46, 47, 66). Astaxanthin, a tetraterpene, is an antioxidant with anti-inflammatory properties; therefore, it is used as a therapeutic agent in atherosclerotic cardiovascular disease (48). In addition, carotenoids are lipid-based antioxidants in the diet that have demonstrated both anti-inflammatory and antibacterial effects (49). The antioxidative effects of phytochemicals have been more widely reported than the antimicrobial activity of triterpenes and sesquiterpenes (41, 46).

Phenolics are secondary metabolites of plants containing one or more hydroxy derivatives of benzene rings and are generally involved in defense against ultraviolet radiation or attacks by pathogens in plants. They are widely distributed in plants and have demonstrated antimicrobial properties (6, 53). For example, there is a wide range of phenolic compounds in bilberry, cranberry, and blueberry, such as anthocyanidins, delphinidin, flavone, luteolin, flavonols, myricetin, quercetin, rutin, and (–)-epigallocatechin-3-gallate (50, 52). Only a few studies have examined the antibacterial properties of anthocyanins. Anthocyanins and cyanidins have been shown to inhibit the growth of *E. coli* (51). Although flavonoids have a very broad spectrum of pharmacological activities, their mode of action is only partially understood. 3-p-Trans-coumaroyl-2-hydroxyquinic acid (CHQA) has shown notable antioxidant activity and antimicrobial activity against *S. aureus* (67). CHQA contains 2-hydroxyquinic acid moiety together with a p-coumaric acid, but it is unclear which moiety or group of this phytochemical is the critically active factor for its antibacterial activity (67). p-Coumaric acid shows antibacterial activity against *E. coli* by destroying cell membranes and binding to the bacterial genomic DNA to inhibit cellular functions (68).

In a previous study, we found that 4-hydroxybenzaldehyde as an adjuvant, which cannot be used as carbon source in *A. baumannii*, showed synergistic effects with only amphenicol antibiotics by increasing the antibiotic influx through a 4-hydroxybenzoate transporter (69). In particular, capsaicin is an active component of *Capsicum* plants (chili peppers) used as food ingredients and has been used for medicinal purposes since ancient times. Owing to its various pharmacological properties, including anti-cancer and beneficial cardiovascular and gastrointestinal tract effects, capsaicin has recently received attention to focus research into its antibacterial and anti-viral effects (55). Its antibacterial effects have been reported against several foodborne pathogens, such as *S. aureus, Helicobacter pylori*, and *P. aeruginosa* (55, 56). Intestinal bacteria can perform reactions that transform more complex plant phenols into simple phenol metabolites (51). In addition, phenolic acid can control the number of microorganisms in the intestine. Gallic acid and caffeic acid have been reported to suppress the growth of *Clostridium* and *Bacteroides* species in intestinal microbiome (51). In fact, phytomedicines, in particular *Triticum aestivum* L. and *Theobroma cacao* L., have been used in zoos to treat gastrointestinal pain and diarrhea in African elephants (*Loxodonta africana*) and crab-eating macaques (70). Especially, cocoa beans have historically been used as a treatment for diarrhea, and the mode of action of cocoa-related flavonoids, i.e., inhibition of salt and water secretion, has been demonstrated *in vitro* (57). The effective use of plant extracts with antimicrobial, antioxidant, and immune-boosting effects in combination with antibiotics can work synergistically and may provide a solution for management of antibiotic-resistant bacteria in veterinary science.

## Concluding remarks

Having used antibiotics for decades, mankind has made tremendous advances in medicine, including veterinary medicine. However, the increase in drug resistance in pathogens due to overuse and misuse of antibiotics has increased both treatment costs and failures. The WHO, the World Animal Health Organization, and the Food and Agriculture Organization of the United Nations are attempting to address the worldwide One Health problem (1). Research should continue to identify new natural substances that exhibit antimicrobial and anti-inflammatory activity against multi-drug resistant bacteria without adverse effects on animals. A new mechanistic discovery of the action of phytochemicals will allow a better understanding of their pharmacological effects, insights into their medicinal potential, and strategies to discover drugs from plant sources. Thus, these refined or extracted phytochemical compounds could be potential candidates for improving the treatment of zoonotic diseases in both humans and animals. Further research including clinical trials should be performed to obtain more comprehensive knowledge of the use of phytochemicals as drugs in veterinary medicine.

## Author contributions

BS drafted and wrote all parts of the manuscript. WP provided substantial modifications and conceptual advice at all stages of manuscript preparation.

### Conflict of interest statement

The authors declare that the research was conducted in the absence of any commercial or financial relationships that could be construed as a potential conflict of interest.

## Footnotes

^1^CDC https://www.cdc.gov/salmonella/index.html
